# The Effect of Rosavin, a Characteristic Compound of *Rhodiola rosea*, on BMP-2 Induction and Osteoblast Proliferation In Vitro

**DOI:** 10.3390/ijms26136075

**Published:** 2025-06-24

**Authors:** Piotr Wojdasiewicz, Edyta Wróbel, Krzysztof Stolarczyk, Elżbieta U. Stolarczyk, Agnieszka Mikulska, Dariusz Szukiewicz

**Affiliations:** 1 Department of Biophysics, Physiology and Pathophysiology, Faculty of Health Sciences, Medical University of Warsaw, 5 Chałubińskiego Street, 02-004 Warsaw, Poland; edyta.wrobel@wum.edu.pl (E.W.); agnieszka.mikulska@wum.edu.pl (A.M.); dariusz.szukiewicz@wum.edu.pl (D.S.); 2 Faculty of Chemistry, University of Warsaw, 1 Pasteura Street, 02-093 Warsaw, Poland; kstolar@chem.uw.edu.pl; 3 Spectrometric Methods Department, National Medicine Institute, 30/34 Chełmska Street, 00-725 Warsaw, Poland; e.stolarczyk@nil.gov.pl

**Keywords:** rosavin, human osteoblasts, osteoporosis, adaptogens, golden root, bone morphogenetic protein 2, cell cultures, phyto-compounds, nutrition

## Abstract

Rosavin, a glycoside isolated from *Rhodiola rosea*, exhibits various biological activities, including potential modulation of metabolic pathways. Despite promising findings in animal models, its effects on many human bone cells remain unexplored. This study aimed to investigate, for the first time, the in vitro effects of rosavin on human osteoblasts (HOBs), focusing on BMP-2 expression, cell morphology, and culture confluence as indicators of osteogenic activity. HOB cultures were treated with 50 µM or 100 µM rosavin for 21 days. BMP-2 expression was measured by ELISA, collagen production was assessed via Sirius Red staining, and cell morphology and confluence were evaluated using phase-contrast microscopy. A significant increase in BMP-2 expression was observed in the 100 µM rosavin group compared to the mineralization control (*p* < 0.05), particularly on days 14 and 21. Both rosavin-treated groups exhibited higher confluence than controls, with the 50 µM group showing unexpectedly greater confluence than the 100 µM group. Rosavin at 50 µM also promoted a cuboidal morphology characteristic of active HOBs. The presence of collagen validated both the successful progression of the mineralization process and the correct implementation of the experimental protocol. Rosavin enhances BMP-2 expression and supports HOB proliferation and morphological maturation in vitro. These findings suggest its potential as a supportive agent in the prevention or treatment of metabolic bone diseases. Further research is necessary to determine its bioavailability, safety profile, and therapeutic relevance in clinical settings.

## 1. Introduction

Rosavin is a plant-derived glycoside primarily isolated from *Rhodiola rosea* (golden root), a member of the Crassulaceae family. Its chemical formula is C_20_H_28_O_10_, with a molecular mass of 428.43 g/mol. Compounds of this class have long attracted scientific interest due to their potential pharmacological properties, including anti-inflammatory, anticancer, neuroprotective, and adaptogenic effects [1,2]. Extracts of *Rhodiola rosea* are widely used as dietary supplements to support the body under conditions of stress, fatigue, cognitive impairment, weakened immunity, and to enhance both physical and mental performance [3,4]. In several countries, rosavin is administered orally in extract form, and in Sweden, it is utilized in children to improve concentration and reduce fatigue [5]. Contemporary research on the pharmacological potential of rosavin suggests its significant role in modulating metabolic processes, including those related to bone tissue homeostasis [6,7].

Bone tissue, in addition to its structural functions, constitutes a dynamic system involved in metabolic and homeostatic processes. Specifically, the regulation of bone formation and resorption occurs through the coordinated activity of osteoblasts and osteoclasts, with cytokines and growth factors playing a crucial role in these mechanisms [8]. One of the most important regulators of osteogenesis is bone morphogenetic protein 2 (BMP-2), which participates in the differentiation of progenitor cells into osteoblasts, supports matrix mineralization and facilitates reparative processes [9,10]. BMP-2 is of critical importance in regenerative therapy and in the treatment of bone disorders, including osteoporosis and non-healing fractures [11]. Osteoporosis, a disease characterized by progressive loss of bone mass and increased fracture risk, represents a significant global health issue. It is estimated that over 200 million individuals worldwide suffer from osteoporosis, with approximately 10 million cases in the United States alone [12,13]. Growing awareness of the significance of bone metabolism and its molecular regulation encourages the search for novel, effective therapeutic strategies to support bone tissue regeneration and prevent its degradation. In this context, the hypothesis emerges that rosavin may influence BMP-2 expression in human osteoblasts (HOBs), thereby opening new therapeutic possibilities for the treatment of osteoporosis and other metabolic bone diseases [14,15,16].

The aim of this study is to conduct the first-ever assessment of the effects of rosavin on BMP-2 expression in HOB cultures in vitro. Additionally, the assessment will include the estimation of cultures morphology and density following rosavin treatment.

Although previous studies in animal models have suggested that rosavin may influence bone tissue metabolism, its direct effects on HOBs have not been sufficiently investigated. Therefore, the aim of the present study was to determine whether rosavin exerts a direct effect on human osteogenic cells and to identify the potential molecular mechanisms underlying this action. The outcomes of this study may serve as a basis for further exploration of rosavin’s therapeutic utility, especially regarding osteoporosis and related bone metabolic disorders. Moreover, they may support the advancement of innovative approaches in regenerative medicine aimed at bone tissue regeneration.

## 2. Results

### 2.1. Assessment of BMP-2 Expression in Osteoblast Cultures

**Figure 1:**
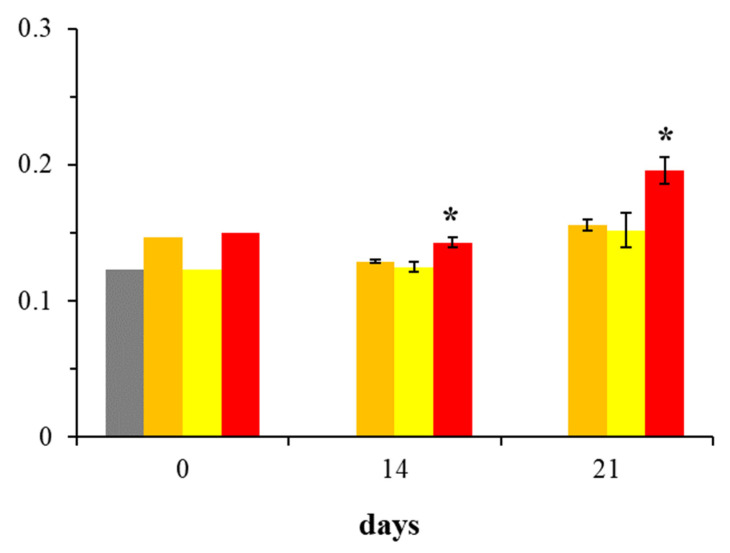
The graph illustrates the changes in mean BMP-2 expression, measured by absorbance according to ELISA assay guidelines (Abcam, ab277085), throughout the experiment at different time points (0, 14 and 21 days), depending on the applied cell culture conditions and rosavin supplementation. The groups are color-coded as follows: (●) gray represents DMEM g, (●) orange represents DMEM m, (●) yellow represents R50, and (●) red represents R100. Data are presented as mean ± standard deviation (SD). Asterisks (*) indicate statistical significance at *p* < 0.05.

The graphical representation of BMP-2 expression changes in HOBs across all groups throughout the experiment is presented in Figure 1. At the beginning of the experiment (day 0), no difference in BMP-2 expression was observed between the DMEM m and DMEM g groups. As the study progresses, an increase in BMP-2 presence can be observed in the R100 group compared to other cell cultures.

**Figure 2:**
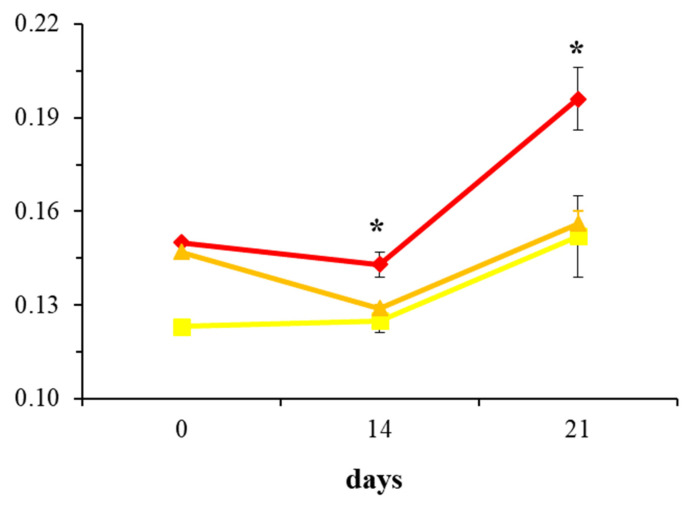
Time points of 0, 14, and 21 days in relation to BMP-2 expression, comparing DMEM m (●), R50 (●) and R100 (●), are presented in a line graph. Data are expressed as mean ± standard deviation (SD). Asterisks (*) indicate statistical significance at *p* < 0.05.

Figure 2, utilizing a line graph for visualization, further emphasizes the differences in BMP-2 expression among the DMEM m, R50, and R100 groups across the selected experimental days. Notably, treatment with 100 µM rosavin (R100) significantly increased BMP-2 expression on days 14 and 21 compared to the mineralization medium control (DMEM m) (*p* < 0.05). In contrast, 50 µM rosavin (R50) did not exhibit significant differences in BMP-2 expression relative to DMEM m. Comparing the 14th and 21st day of the effect of 100 µM rosavin on the osteoblast culture, a 37% increase in BMP-2 presence was observed in the osteoblast culture on day 21 compared to day 14. In summary, 100 µM rosavin effectively enhances BMP-2 expression in human osteoblast cultures in vitro, with effects becoming evident from day 14 and intensifying by day 21.

### 2.2. Evaluation of Collagen Production in Osteoblast Cultures

**Figure 3:**
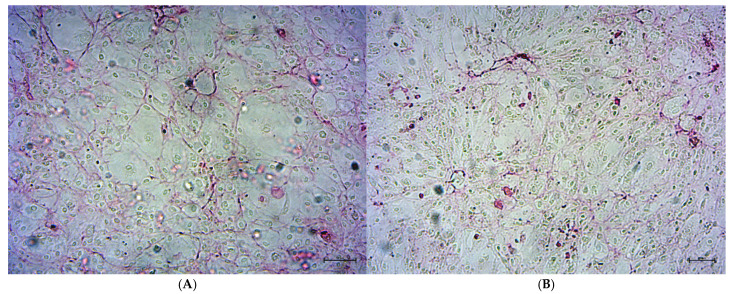
Localization of collagen during differentiation of HOBs on days 14 (**A**) and 21 (**B**). Purple staining was observed in collagen, an extracellular matrix protein that provides a substrate for the mineralization process. Independent experiments were performed, and representative images are shown. Scale bar: 50 μm (day 14); 20 μm (day 21).

Sirius Red staining revealed the presence of collagen as early as day 14 in DMEM m (Figure 3), indicating the initiation of mineralization and osteoblast maturation, which is consistent with previous studies [17,18,19]. The collagen content remained stable up to day 21 of observation.

### 2.3. Analysis of Osteoblast Confluence in Culture

**Figure 4:**
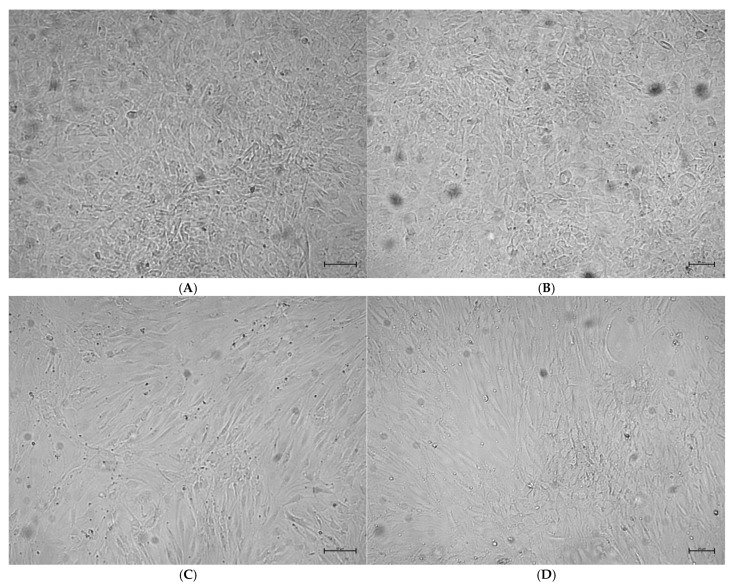
Morphological changes and confluence of the HOBs during their in vitro differentiation treated with 50 μM (**A**,**B**) or 100 μM (**C**,**D**) rosavin. Phase contrast images were taken on day 14 (**A**,**C**), and 21 (**B**,**D**) of culture. In HOB cultures treated with 50 µM rosavin, all cells are cuboidal, larger, and the confluence reaches 100% (**A**). Similar observations were made on day 21 of cultures treated with 50 µM rosavin (**B**). In cultures treated with 100 µM rosavin, most cells had an elongated fibroblast-like morphology on day 14 of culture. Areas of dense cells with a cuboid shape were observed (**C**). The morphology of the cells on day 21 of culture was similar to that on day 14 of culture, HOB cells with a fibroblast-like shape and larger areas of cells with a cuboid shape in high density (**D**). It was observed that the confluence of the HOB cells treated with 100 μM (**C**,**D**) was lower than that of those treated with 50 μM (**A**,**B**). Independent experiments were performed, and representative images are shown. Scale bar: 50 μm (day 14); 20 μm (day 21).

**Figure 5:**
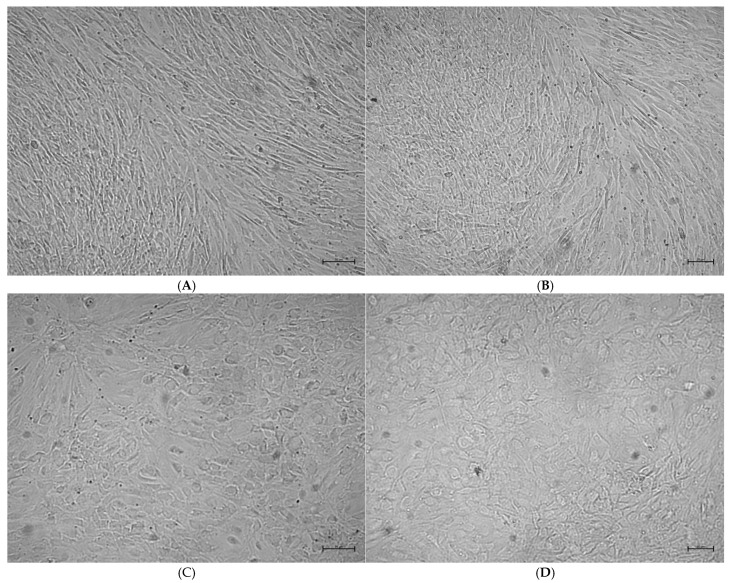
Morphological changes of the HOBs during their in vitro proliferation and differentiation. The cells were cultured in growth (**A**,**B**) or mineralization medium (**C**,**D**). Phase contrast images were taken on day 14 (**A**,**C**), and 21 (**B**,**D**) of culture. The morphology of the HOBs changed from a fibroblastic-like cultured with growth medium compared to a more cuboidal shape cultured with mineralization medium. An increase in confluency was observed in HOB cultures on day 21 compared to day 14 of culture. Independent experiments were performed, and representative images are shown. Scale bar: 50 μm (day 14); 20 μm (day 21).

Microscopic evaluations demonstrated that on days 14 and 21, rosavin-treated groups (R50 and R100) exhibited higher osteoblast confluence compared to DMEM g and DMEM m. The highest confluence was observed on day 21 in the R50 group, followed by the R100 group, DMEM m, and the lowest in DMEM g (Figure 4 and Figure 5). In control cultures without rosavin treatment, osteoblasts showed an elongated fibroblast-like shape both on day 14 and 21 of culture. In the DMEM groups, the cell shape became less cuboidal, and the cells appeared more flattened and larger. The confluence compared to 50 µM rosavin is lower in the culture in the differentiation medium without rosavin treatment.

## 3. Discussion

To the best of the authors’ knowledge, this is the first study to investigate the effects of rosavin on HOBs in vitro, with the aim of evaluating its potential in the treatment or prevention of metabolic bone diseases. This research is highly relevant given the global rise in such conditions, particularly osteoporosis, driven by aging populations, unhealthy lifestyles, environmental stressors, and side effects of treatments for unrelated diseases such as COPD, asthma, and autoimmune disorders [20,21,22,23].

The increasing prevalence of metabolic bone diseases poses a significant burden on healthcare systems due to prolonged work incapacity, disability, and mortality. Thus, identifying new therapeutic strategies is essential not only from a biological and ethical standpoint but also from a socioeconomic perspective. This study introduces a novel approach, focusing on rosavin—a compound already marketed as a dietary supplement in several countries—for individuals under environmental or psychological stress, intense physical exertion, or seeking metabolic support [24,25,26].

Rosavin, alongside salidroside, is one of the primary bioactive constituents of *Rhodiola rosea*, known for its health-promoting properties [27,28,29]. Although various studies have explored rosavin’s effects on different physiological systems—primarily in rodent models—limited data exist on its impact on the musculoskeletal system, particularly regarding bone regeneration, mineralization, and resorption [30,31,32,33]. To date, no studies have examined its effects on human bone cells, even under controlled in vitro conditions.

In response to this gap, the present study investigates rosavin’s influence on human osteoblast metabolism in vitro, specifically examining BMP-2 expression and the confluence of HOB cultures.

According to the authors, evaluating the presence of BMP-2—a protein known to stimulate osteoblast proliferation and promote bone mineralization (with an anti-osteoporotic effect) [34,35,36,37]—and assessing the confluence of cultures treated with rosavin represents a small yet significant initial step in determining the potential therapeutic relevance of this compound for metabolic bone diseases.

The results indicate that rosavin, at a concentration of 100 μM, when added to a medium stimulating osteoblast mineralization and differentiation (R100 group), significantly increases BMP-2 expression compared to the corresponding control group (DMEM m). This effect was evident on both day 14 and day 21, the final day of the experiment (Figure 1 and Figure 2). Interestingly, the lower concentration of 50 μM (R50 group) did not result in statistically significant differences relative to the DMEM m group.

It is important to emphasize that the choice of 50 μM and 100 μM concentrations in this study was based on an informed yet ultimately arbitrary decision by the researchers. This approach stemmed from prior preliminary trials, in which considerably lower concentrations of rosavin—such as 1 μM, 5 μM, and 10 μM—had been tested. Those lower doses failed to produce any measurable changes in BMP-2 expression or cell confluence in HOB cultures.

As a result, the rosavin concentration was increased by approximately 5- to 10-fold relative to doses previously used in rodent studies [31,32]. This adjustment yielded a favorable outcome, though only at the higher concentration of 100 μM (R100). The findings suggest that this dose may represent a critical threshold capable of enhancing BMP-2 synthesis in HOBs. This observation is of particular importance for future investigations, especially in the context of determining an effective therapeutic dose that could achieve sufficient bioavailability within bone tissue.

Data from the first day of the experiment (day 0) (Figure 1) allowed the assessment of whether both cultures started with similar levels of BMP-2 expression after the introduction of the new medium for the DMEM m group. In this context, the DMEM g group acted as a reference control for the DMEM m group. The absorbance values for BMP-2 in DMEM g were expected to be comparable to those in DMEM m. Based on the available experimental data, it can be concluded that the differences in BMP-2 expression between DMEM g and DMEM m on day 0 are small, which was an expected result justifying the continuation of further analyses.

Another important finding in this study was the presence of collagen in the DMEM m cell cultures, which was crucial for self-validation of the accuracy of laboratory procedures and the proper functioning of the applied differentiation media. To ensure that the mineralization medium used in the DMEM m group, as well as in the R50 and R100 groups, functioned correctly according to its intended purpose and that the purchased HOB cultures responded predictably and in line with their expected physiological maturation, photographic documentation was used to confirm the presence of collagen on days 14 and 21 (Figure 3). Collagen, directly associated with bone tissue mineralization, serves as a key indicator of the completion of this process [38,39,40]. The results confirmed that mineralization had already occurred in the DMEM m group by day 14, which was consistent with findings reported by other researchers [17,18,19]. Furthermore, on day 21, the mineralization process remained evident and had progressed as expected. These findings confirmed that the purchased HOB cultures, as well as the mineralization and differentiation media (used not only in the DMEM m group but also in the experimental R50 and R100 groups), were functioning correctly, responded predictably, did not exhibit any pathological or adverse effects on days 14 and 21, and that laboratory procedures were carried out with the necessary diligence and professionalism.

In addition to the effect of rosavin on BMP-2 secretion by HOBs, its influence on cell morphology was also investigated to assess whether visible changes accompanied the cytochemical response. As shown in Figure 4 and Figure 5, HOB cultures from all four experimental groups (DMEM g, DMEM m, R50, and R100) were evaluated on days 14 and 21 with respect to confluence and cell shape. Both rosavin-treated groups (R50 and R100) exhibited markedly higher confluence than the control groups, with the R50 group showing the highest density—despite its lower rosavin concentration.

These findings suggest that rosavin enhances HOB proliferation, which may be of particular relevance in the context of osteoblast deficiency and unbalanced bone remodeling. The increased confluence observed in R50, and to a lesser degree in R100, appears to result from complex, multifactorial actions of rosavin rather than a simple dose-dependent relationship with BMP-2 expression. While BMP-2 absorbance was highest in the R100 group, this did not translate into greater confluence, suggesting other regulatory mechanisms are involved.

It is possible that higher concentrations of rosavin induce feedback inhibition or downregulation of key mitogenic pathways [41,42,43], leading to reduced cell division. Such mechanisms, if dysregulated, might even carry carcinogenic potential under pathological conditions.

Microscopic images also revealed morphological differences. In the DMEM g group, cells exhibited predominantly fibroblast-like shapes, while those in the DMEM m and R50 groups showed a more cuboidal morphology, characteristic of actively differentiating osteoblasts [44]. The R100 group displayed a mixed morphology, including fibroblast-like cells, which are typically associated with lower metabolic activity and structural support functions [45]. This morphological pattern aligns with the reduced confluence observed in R100 and supports the hypothesis of a concentration-dependent inhibitory feedback at higher rosavin doses.

It should be remembered that the current experiment focused solely on selected bone tissue cells—HOBs—and not on whole bone tissue. Although, on one hand, the observed confluence and morphology in the R100 group may seem somewhat disappointing (since the R50 group exhibited greater and more expected responsiveness in terms of both proliferation and morphology), it should be noted that healthy bone tissue also contains osteoclasts and immune cells. Therefore, the occurrence of a self-limiting effect of rosavin at concentrations above 100 μM might, in the long term, be more beneficial, as it may reduce the risk of undesired activation or overdevelopment of the osteoclastic lineage, as observed with other compounds [46,47].

This finding underscores the need for further research into the specific cellular pathways and transcription factors modulated by rosavin that may contribute to the acceleration of osteoblast proliferation. Although increased confluence—particularly in the R50 group—may suggest therapeutic potential for metabolic bone disorders, its relevance and safety in oncological settings warrant careful consideration. Increased cell proliferation is not necessarily a favorable outcome in cancer-related scenarios. Therefore, it is crucial to continue research on rosavin’s effects on cell division to determine whether this stimulation is regulated by environmental and intracellular mechanisms or whether it may lead to uncontrolled cell proliferation and potential tumorigenesis. Considering current scientific reports, it is worth to mention that phytochemicals are most commonly associated with carcinoprotective activity [48,49].

As previously noted, the current findings represent an initial step in evaluating rosavin’s potential therapeutic benefits in the prevention and treatment of metabolic bone diseases. Further comprehensive studies are required to validate and expand upon these results. Future experiments could be enhanced by directly assessing mineralization progression and collagen deposition in the R50 and R100 groups. Additionally, employing complementary techniques—such as alkaline phosphatase (ALP) activity assays—may provide deeper insights into osteoblast function.

To better understand rosavin’s impact on cell proliferation, future studies should also include direct cell counting in stained HOB cultures, accompanied by appropriate statistical analysis. Despite its preliminary nature, this study is the first to investigate rosavin’s effects on HOBs and lays a valuable foundation for future work.

Given rosavin’s status as a well-tolerated phytopharmaceutical with documented systemic benefits [25,26], its potential role in bone health warrants continued investigation. Its oral administration offers practical advantages [50,51,52] but also highlights the need for pharmacokinetic studies to determine its bioavailability, metabolic stability, and safe, effective dosing in the context of bone metabolism.

Importantly, this study was conducted using HOBs derived from a healthy donor. Future research should extend to osteoblasts from patients with metabolic bone diseases such as osteoporosis, rheumatoid arthritis, Paget’s disease, osteogenesis imperfecta, or steroid-induced bone loss. Rosavin’s effects in these pathological contexts may differ and remain unpredictable. Nevertheless, the promising results presented here strongly support continued research into rosavin as a potential adjunct in bone health strategies, particularly given the ongoing lack of novel and broadly effective therapies for skeletal disorders.

## 4. Materials and Methods

### 4.1. Culture of Human Osteoblasts Line

HOBs were derived from primary human osteoblasts isolated from trabecular bone tissue of the femur around the knee or hip joint (C-12720). Cultures of HOBs were placed in a 25 cm^2^ cell culture flasks, containing the standard culture growth medium, including Basal Medium (PromoCell, Heidelberg, Germany, C-27001) and SupplementMix (C-39615), which was supplemented with a 1% antibiotic–antimycotic mixture consisting of 10.000 U of penicillin and 10 mg of streptomycin (Sigma-Aldrich, St. Louis, MO, USA, P0781). The cultures were cultured in an incubator at 37 °C in 5% CO_2_ and 95% humidified atmosphere. The medium was changed every 3 days. After confluence, the HOBs were detached using the DetachKit (PromoCell, C-41200), which consists of three components: HEPES BSS (HEPES buffered Balanced Salt Solution), Trypsin/EDTA Solution and TNS (Trypsin Neutralization Solution). The DetachKit comes with a Trypsin/EDTA ratio of 0.04%/0.03%. The cells from the sixth passage were used in all of the experiments. Cells were seeded in 24-well dishes at a density of 44 × 10^3^ cells per well (1.9 cm^2^) in a growth medium. The culture medium was changed every 3 days until the cells reached 70–80% confluence. When confluence was achieved, four experimental groups were formed:

1. HOBs containing the osteoblast growth medium, including Basal Medium and SupplementMix (DMEM g).
2. HOBs containing the osteoblast mineralization medium (PromoCell, C-27020) and SupplementMix (C-39616) (DMEM m).
3. HOBs containing the osteoblast mineralization medium, SupplementMix, and 50 µM rosavin in final dilution (R50).
4. HOBs containing the osteoblast mineralization medium, SupplementMix, and 100 µM rosavin in final dilution (R100).

Cell cultures were performed for 21 days, and the medium appropriate for the research groups was changed every 3 days.

The morphology of the cells was analyzed using an inverted, phase-contrast microscope (Zeiss Primovert, Jena, Germany) on days 14 and 21 of culture. Images were recorded with a Zeiss Axiocam ERc 5s camera for all cell culture variants.

### 4.2. Preparation of Rosavin Solution

Rosavin (Sigma-Aldrich, SML0336), in powder form, was dissolved in sterile PBS (ThermoFisher, San Diego, CA, USA, 14190169) to obtain a stock concentration of 10 mM. Appropriate volumes of the solution were then added to the cell cultures to obtain final rosavin concentrations of 50 µM or 100 µM.

### 4.3. Enzyme-Linked Immunosorbent Assay

Cells were seeded in 24-well plates and cultured for 21 h. Supernatants were collected 72 h after rosavin treatment of the cultures (R50 and R100) at three time points, 0, 14 and 21 days of culture. Both rosavin-treated and nontreated supernatants were stored at −20 °C. Commercial Enzyme-linked immunosorbent assay (ELISA) kits (Abcam, Waltham, MA, USA, ab277085) were used for the detection of the BMP-2 level in the cell supernatants. Enzyme plates were prepared with standard, blank, and sample wells. Then 50 μL of standard or sample was added to the appropriate wells. After adding 50 μL of Antibody Cocktail to all wells, the wells were incubated at room temperature for 1 h. After this time, each well was washed three times with 350 μL of 1X Wash Buffer. A total of 100 μL of TMB Development Solution was added to each well and incubated for 10 min. Finally, 100 μL of Stop Solution was added and the change in OD at 450 nm was analyzed using a microplate spectrophotometer (ASYS UVM 340 Microplate Reader, Biocompare, San Francisco, CA, USA).

### 4.4. Determination of Collagen Production and Cell Confluence in Cell Cultures

The collagen assay is based on the binding of Sirius red dye to the collagen triple helix. Sirius red staining was performed in cell cultures containing osteoblast mineralization medium and SupplementMix (DMEM m). On day 14 and 21 of culture, cells were washed three times with PBS calcium magnesium (ThermoFisher, 14040174), then 0.1% Sirius red (Sigma-Aldrich, 365548) was added and incubated for 60 min at room temperature. Next, the plates were washed three times with 10 mM HCl. The presence of collagen was observed under an inverted microscope (Primovert). A similar approximate assessment and comparative analysis were performed to evaluate the confluency of cell cultures in the DMEM m, R50, and R100 groups on days 14 and 21. This analysis aimed to determine the potential impact of rosavin on HOB cell proliferation.

### 4.5. Bioethical Issues

The research was conducted on purchased, commercialized HOBs and does not require the consent of the Bioethics Committee in accordance with the international guidelines of the Helsinki Declaration [53] and the International Society for Stem Cell Research (ISSCR) [54], because it does not include biological material obtained from living human donors or any procedures affecting human subjects.

### 4.6. Statistical Analysis

Differences in BMP-2 expression levels between the studied cell culture groups were analyzed to produce descriptive statistics for all experimental groups. The normality of the distribution of experimental results was assessed by the Shapiro–Wilk test. The homogeneity of variances was evaluated by the Brown–Forsythe test. On this basis, the Kruskal–Wallis test was used to investigate the significance of differences between the tested groups. The results were considered to be statistically significant when *p* values were < 0.05.

## 5. Conclusions

This study is the first to demonstrate the effects of rosavin on HOBs in vitro, showing that a 100 μM concentration significantly increases BMP-2 expression, while both 50 μM and 100 μM concentrations enhance cell confluence. Notably, the lower dose yielded greater confluence, suggesting a possible self-regulatory mechanism at higher concentrations. Rosavin (R50) also promoted a cuboidal osteoblast morphology, consistent with active, differentiating cells. These findings indicate that rosavin may support osteoblast proliferation and bone-forming activity, highlighting its potential as a complementary agent in managing various metabolic bone diseases, such as osteoporosis, as well as complications of bone injury treatment, including delayed union and nonunion. However, further research is warranted to assess its bioavailability, optimal dosing, long-term safety, and efficacy in diseased HOB models.

## Data Availability

The datasets generated in this study are available from the corresponding author upon reasonable request.
